# Dopaminergic foundations of personality and individual differences

**DOI:** 10.3389/fnhum.2014.00874

**Published:** 2014-10-30

**Authors:** Luke D. Smillie, Jan Wacker

**Affiliations:** ^1^Melbourne School of Psychological Sciences, The University of MelbourneMelbourne, VIC, Australia; ^2^Department of Psychology, University of HamburgHamburg, Germany

**Keywords:** dopamine, personality, extraversion, schizotypy, reward

For several years, theory and research in Personality Neuroscience has linked dopamine function with various aspects of personality and individual differences. This literature builds on research in basic neuroscience concerning the role of dopamine in behavior and experience, with the aim of understanding the ways in which this neurotransmitter system influences *regularities* in behavior and experience. We organized this special issue on “Dopaminergic Foundations of Personality and Individual Differences” with the goal of illuminating the diversity of roles that dopamine plays in personality and individual differences. To introduce this topic, we provide a brief sketch of the current understanding of the functions of the dopamine system. In doing so, we place the diverse contributions to this research topic in the context of this rich, evolving literature.

## What role does dopamine play in behavior and experience?

The dopamine system can be divided into several anatomically defined branches or pathways. The nigrostriatal pathway (projecting from the substantia nigra to the striatum) is involved in motor control, and has long been of interest in the context of Parkinson's Disease and its therapeutic management via dopamine replacement (see Cenci, 2007). It was initially thought that motor control was the primary or even sole function of dopamine (e.g., Koob, 1982). However, this perspective has given way to a reward-processing interpretation of dopamine, focussed primarily on the mesolimbic pathway (projecting from the ventral tegmental area to limbic and forebrain areas including the striatum) (Robbins and Everitt, 1996; Wise, 2004; Schultz, 2007). One early theory helped integrate these diverging perspectives by proposing that the ventral striatum, a target of both nigrostriatal and mesolimbic dopamine, was responsible for converting motivation (i.e., to approach desire goal states) into action (Mogenson et al., 1980).

The reward-processing functions of dopamine have been discussed in terms of motivation by reward, enjoyment of reward, and learning from reward—or “wanting,” “liking” and “learning” (Berridge et al., 2009). Initially it was theorized that dopamine mediated reward “liking”—the hedonic impact of rewarding stimuli (Wise, 1982), and that these pleasure responses sustained reward-directed behavior. This theory enjoyed widespread influence for some time, and explains why dopamine was popularly dubbed “the pleasure chemical,” but has now been abandoned (Wise, 2004). One critique came from the addiction literature, which showed that dopamine-mediated escalation of drug dependence is accompanied by *decreased* pleasurable responses to those drugs (Robinson and Berridge, 2003). This favors the theory that dopamine mediates motivational “wanting” of reward by conferring stimuli with “incentive salience”—the process through which stimuli become motivationally attractive (Robinson and Berridge, 2003; Berridge et al., 2009). Dopamine is also thought to be responsible for reward learning, with phasic dopamine activity providing the “teacher” signal hypothesized in reinforcement learning models (Schultz et al., 1997; Schultz, 2007). Although reward wanting theories appear compatible with reward learning theories, they have not yet been integrated into a cohesive theoretical framework (see Alcaro et al., 2007).

Dopamine also has a major role in cognitive function and dysfunction. The mesocortical dopamine pathway (projecting from the ventral tegmental area to the dorsolateral prefrontal cortex and the anterior cingulate cortex) is implicated in higher cognitive functions such as working memory and decision-making (Robbins et al., 1996; Arnsten, 1998; Floresco and Magyar, 2006). Although these appear strikingly different to the motivational functions of the mesolimbic dopamine system, mental representations and operations seem likely to facilitate motivated action. That is, the mesocorticolimbic dopamine pathways may jointly coordinate the *“anticipation of reward and activation of representations in the PFC needed to achieve it”* (Miller and Cohen, 2001, p. 182). The higher cognitive functions of dopamine have implications for creative behavior, which is typically operationalized using tests of cognitive flexibility and divergent thinking. Ashby et al. (1999) suggest that this may explain the apparent impact of induced positive affect on creativity; positive affect is often preceded by reward delivery, which will often stimulate dopamine release. Finally, an enduring theory has posited a central role for dopamine in the cognitive disturbances seen in schizophrenia (e.g., Gray et al., 1991). A later iteration of this theory has related *mesocortical* dopamine to cognitive deficits (e.g., executive dysfunction) and negative symptoms (e.g., anhedonia), and *mesolimbic* dopamine to positive symptoms (e.g., hallucinations and delusions) (Lindenmayer et al., 2013).

This brief overview is only intended to orient the reader, illustrate the breadth of processes to which dopamine has been linked, and thereby foreshadow the diversity of topics addressed in this special issue. For more in-depth perspectives on dopamine function the interested reader is encouraged to consult the references cited here.

## What role does dopamine play in personality and individual differences?

### Extraversion and reward-processing

Perhaps the earliest and most influential perspective on the role of dopamine in personality was Gray's (1973) suggestion that dispositional variation in the reward-processing functions of the dopamine system would likely manifest as a major, to-be-identified personality dimension. This dimension was later identified as extraversion (Depue and Collins, 1999), an enduring proposal that is currently the dominant neurobiological perspective on this trait (see Smillie, 2013), and has motivated over one-third of the contributing articles to this special issue.

Our first two articles demonstrate that the effects of dopaminergic pharmacological agents are entirely dependent on extraverted personality: *Depue and Fu* observe contextual facilitation of incentive motivation processes in extraverted individuals for whom a contextual ensemble was paired with a dopamine agonist. These findings appear to link extraversion with the dopamine-driven processes that associate contexts with reward. *Chavanon and colleagues* demonstrate dose-dependent effects of a dopamine antagonist on an EEG-recorded neural activity—localized to prefrontal regions innervated by the mesocorticolimbic dopamine pathway)—and that these effects are diametrically opposed for extraverted and introverted individuals. These findings are potentially explained in terms of extraversion-related individual differences in pre- and post-synaptic responsivity to the dopamine antagonist.

Our next two articles relate extraversion to EEG-derived indices of reward system activity: *Cooper and colleagues* replicate their recent finding that extraversion is associated with a neural index of the dopaminergic teacher signal specified in models of reinforcement learning, and show that this generalizes to a conceptually related trait concerning reward anticipation. *Knyazev* shows that, in more extraverted individuals, there is a relation between self-referential thoughts and alpha power in the posterior hub of the Default Mode Network—a resting state network that has been implicated in self-centered cognition, and which appears to have a basis in dopaminergic neurotransmission.

A further two articles focussing on extraversion and reward-processing employ computational models of reward learning: *Pickering and Pesola* identify a number of specific parameters within biologically-plausible models of reward learning that potentially represent the neural substrates of traits such as extraversion (e.g., those that modulate the strength of the neuroplastic effects of phasic dopamine cell firing). *Skatova and colleagues* model extraversion-related differences on a reinforcement learning task in terms of two distinct forms of learning that are difficult to dissociate in typical studies of this kind. After discarding participants who were not engaged with the learning task, they found that extraversion is related to error-driven learning processes, distinct from other learning processes.

### Other reward-related individual differences

Reward-related processes have also been implicated in several individual differences constructs beyond extraversion: *Treadway and colleagues* focus on chronic perceptions of stress, which they find is associated with reduced processing of reward and punishment in the medial prefrontal cortex. *Schultheiss and Schiepe-Tiska* focus on the implicit motive “need for power” (i.e., the tendency to experience power over others as rewarding), which they theorize may have a basis in dopamine-driven learning processes centered on the striatum. *Richter and colleagues* report that the degree to which both monetary rewards and punishments modulate reaction time and BOLD measures of interference processing (i.e., objective indicators of differential reinforcement sensitivity) covaries with the DRD2/ANKK1 TaqIA polymorphism of the dopamine D2 receptor gene. Relatedly, findings by *Kawasaki and Yamaguchi* suggest that the degree to which visual working memory capacity increases for preferred versus non-preferred colors may constitute another useful indicator of reward sensitivity that may be linked to brain dopamine in future work.

The rewarding impact of prosocial actions and outcomes (e.g., Harbaugh et al., 2007) suggests that prosocial behavior may also be linked with dopaminergic reward-processing. In line with this, *Jiang and colleagues* conclude from their qualitative review of the literature that a specific dopaminergic gene variant, the D4 receptor gene exon III (DRD4) polymorphism, influences prosocial behavior depending on environmental influences. *Reuter and colleagues* underscore this conclusion by showing that the extent to which individuals behave fairly in the ultimatum game depends on genetic variants of not only the DRD4 but also the D2 receptor gene (DRD2/ANKK1 TaqIA).

### Cognitive processes

The role that dopamine appears to play in symptoms of schizophrenia has clear implications for individual differences reflecting psychosis-proneness and schizotypy. In the first of two papers on this topic, *Grant and colleagues* report that scores on a German translation of the “Oxford-Liverpool Inventory of Feelings and Experiences” (O-LIFE; Mason and Claridge, 2006) are related to a number of dopamine-related genetic polymorphisms. Conversely, *Ettinger and colleagues* examine the association between two measures of psychosis-proneness and neural activity (fMRI) during procedural learning. Their observed associations with BOLD response in several dopamine-relevant regions, including the striatum, are consistent with dopamine's role in both procedural learning and psychosis-proneness.

As noted in our brief introduction, the role of dopamine in cognitive function also appears to extend to creative problem solving. *Chermahini and Hommel* report a replication of their prior work showing a curvilinear association between spontaneous eye-blink rate (a putative non-invasive marker of central dopamine tonus) and creativity (divergent thinking), with optimal performance at average eye-blink rates (presumably reflecting average levels of central dopamine).

### Theoretical integration

How can we make coherent sense of the variety of individual differences phenomena in which dopamine appears to be involved? In the final article in our special issue, *DeYoung* proposes that the over-arching function of the dopamine system is to promote exploration, which he divides into cognitive exploration (driven by salience-coding dopamine neurons, and linked with trait domains such as openness/intellect) and behavioral exploration (driven by value-coding dopamine neurons, and linked with trait domains such as extraversion). His model provides an elegant framework for integrating the various contributions to this special issue, as well as the broader literature concerning the dopaminergic foundations of personality and individual differences.

### Summary and outlook

The sixteen articles in this special issue are a testament to the significant advances that have been made in personality neuroscience and related fields in recent years. Some of these articles have yielded novel findings, while others have served the important task of replicating and consolidating existing research. Overall, they should leave most readers convinced that dopamine function does play a role in personality and other individual differences. Equally, they demonstrate that there is no simple one-to-one correspondence between the neurotransmitter dopamine and any single personality trait. This has often been noted (e.g., Zuckerman, 2005) but is perhaps tempting to ignore. In recognition of this complexity, a challenge for future research is to develop and evaluate more integrative perspectives concerning the multiple neurobiological bases of so-called dopaminergic traits, and the multiple ways in which dopamine influences regular patterns of behavior and experience.

### Conflict of interest statement

The authors declare that the research was conducted in the absence of any commercial or financial relationships that could be construed as a potential conflict of interest.
